# Histological scoring system for subchondral bone changes in murine models of joint aging and osteoarthritis

**DOI:** 10.1038/s41598-020-66979-7

**Published:** 2020-06-22

**Authors:** Keita Nagira, Yasunari Ikuta, Masahiro Shinohara, Yohei Sanada, Takenori Omoto, Haruhisa Kanaya, Tomoyuki Nakasa, Masakazu Ishikawa, Nobuo Adachi, Shigeru Miyaki, Martin Lotz

**Affiliations:** 1 Department of Molecular Medicine, Scripps Research, La Jolla, California USA; 2 Department of Orthopaedic Surgery, Tottori University, Tottori, Japan; 3 Department of Orthopaedic Surgery, Graduate School of Biomedical & Health Sciences, Hiroshima University, Hiroshima, Japan; 4 Department of Rehabilitation for the Movement Functions, National Rehabilitation Center for Persons with Disabilities, Saitama, Japan; 5 Medical Center for Translational and Clinical Research, Hiroshima University Hospital, Hiroshima, Japan

**Keywords:** Targeted bone remodelling, Cartilage development, Osteoarthritis, Experimental models of disease, Bone

## Abstract

To establish a histopathological scoring system for changes in subchondral bone in murine models of knee osteoarthritis (OA), three key parameters, subchondral bone plate (Subcho.BP) consisting of the combination of Subcho.BP.thickness (Subcho.BP.Th) and angiogenesis, bone volume (BV/TV) and osteophytes, were selected. The new grading system was tested in two mouse OA models, (1) senescence accelerated mouse (SAM)-prone 8 (SAMP8) as spontaneous OA model with SAM-resistant 1 (SAMR1) as control; (2) destabilization of the medial meniscus in C57BL/6 mice as surgical OA model. Results of the spontaneous OA model showed that Subcho.BP.Th was significantly wider, angiogenesis was greater, and BV/TV was higher in SAMP8 than SAMR1. Notably, subchondral bone score was dramatically higher in SAMP8 at 6 weeks than SAMR1, while OARSI cartilage scores became higher only at 14 weeks. In the surgical OA model, the results were similar to the spontaneous OA model, but osteophytes appeared earlier. There were strong correlations both in Subcho.BP.Th and BV/TV between this scoring system and µCT (r = 0.89, 0.84, respectively). Inter-rater reliabilities for each parameter using this system were more than 0.943. We conclude that this new histopathological scoring system is readily applicable for evaluating the early changes in aging and OA-affected murine subchondral bone.

## Introduction

Osteoarthritis (OA) is the most common form of joint diseases with joint pain and activity limitations^1^. OA develops with advancing age in individuals with a combination of risk factor such as obesity, malalignment, female sex and gene polymorphisms^2–6^. Individuals with joint trauma are at risk for onset of OA at younger age^7^. Although OA is a whole joint disease with changes in various joint tissues, including articular cartilage, subchondral bone, synovium and ligaments, the major focus for OA research had remained primarily on the articular cartilage^8^. However, the subchondral bone changes occur at an early phase of the OA process and are closely related to cartilage changes, indicating an important relationship between OA and subchondral bone^9–12^.

Radin *et al*. proposed that increased bone mass and stiffening of the subchondral bone may be a primary event in OA development^13^ and later reported that increases in stiffness of the underlying bone was associated with cartilage degeneration^14^. This was also supported by findings in the Hartley guinea pig where the subchondral bone was mechanically fragile before the onset of cartilage degeneration^15^. Furthermore, according to recent studies, the changes in subchondral bone are considered important features in OA, even in the very early phases of the disease as has been demonstrated in animal models^9,16–20^. Thus, it has been suggested that bone-targeting therapeutic agents such as bisphosphonates^21^, TGFβ signal inhibitor^18^, calcitonin gene-related peptide (CGRP) receptor antagonist^16^, or small molecule inhibitor of the Wnt signal pathway^22^, have potential for OA treatment.

Cartilage changes in murine model are typically assessed on histology with Mankin^23^ or Osteoarthritis Research Society International (OARSI) scoring systems^24^, which capture matrix damage and cellular changes. High-resolution µCT is the most widely used tool to quantitatively capture subchondral bone changes in small animal models^25–27^. However, µCT has limitations in not being able to evaluate cartilage and subchondral bone interaction and not being able to evaluate details such as angiogenesis in subchondral bone. The present study is focused on histological scoring systems for subchondral bone. There were several previous attempts to use histomorphometry to evaluate subchondral bone changes in human tissue samples^28–34^ and in OA animal models^11,35–39^. However, currently, there is no established scoring system that can detect early changes of subchondral bone in murine models. Thus, the purpose of the present study was to develop and validate a semiquantitative histological scoring system that can be applied to the same sections that are commonly used for the evaluation of changes in cartilage or synovium in murine OA models.

## Methods

### Study design

We performed literature review and found four publications about histopathological scoring systems for subchondral bone changes in animal OA models^24,40–42^. A summary of those scoring systems is provided in Table 1. In three out of four of those systems, subchondral bone changes were included with cartilage changes in OA scores. All four papers about subchondral bone scoring recommend Safranin O/fast green (Safranin O) stain. In Jeon’s paper^42^, medial tibial bone sclerosis was scored by measuring the subchondral trabecular bone to marrow ratio. They also measured osteophyte thickness, but this was not scored. Some features in Gerwin’s system^41^ are semi-quantified, but the others are not quantified. In the human OA knee, Aho’s system focuses on subchondral bone changes in much more detail than in animals, but grades are divided into only 0–3, and not applied to a semi-quantified system^43^. Most importantly, the parameters of subchondral bone in OA to be evaluated are not standardized in any of the four systems.

**Table 1:** Comparison of previous histological scoring systems for subchondral bone (SB).

Author	Animal	System	Parameters	Detail of each system
Rudolphi, K. *et al*.^40^	Mouse	0–8	SB thickening	0: normal 3: remodeling process 8: thickening, strong sclerosis
Glasson, S. S. *et al*.^24^	Mouse	0–3	SB thickening	0: normal 1: mild changes 2: severe changes
Gerwin, N. *et al*.^41^	Rat	0–5	SB thickening, marrow, tidemark, calcified caltilage	0–5: calcified cartilage and SB damage (for more information see ref. ^41^.)
Jeon, O. H. *et al*.^42^	Mouse	−5–5	Bone sclerosis*	-5: severe bone loss 0: no increase or decrease 5: severe bone sclerosis

*Medial tibial bone sclerosis was scored by measuring the subchondral trabecular bone to marrow ratio.

To develop a new system, we first defined the tissue compartments in subchondral bone. Then we reviewed histology and µCT literature about mouse models for OA to select the most commonly analyzed subchondral bone parameters that change in OA and can be detected by histology.

For each parameter except for osteophytes, we defined ranges of values that are used to capture OA-related changes. The original values are then converted to a score. In comparison of Subcho.BP.Th, the inclusion of control groups is essential. To test new histological scoring system, we used two OA animal models, 1) senescence accelerated mouse (SAM)-prone 8 (SAMP8)^44–46^ as spontaneous OA model with SAM-resistant 1 (SAMR1) as the control of SAMP8. 2) destabilization of the medial meniscus (DMM)^47^ in C57BL/6 mice as surgical OA model with sham surgery and no surgery as controls. In addition, to validate the histological scoring system, we applied µCT analysis and performed statistical analysis to determine correlations between the systems.

### Definition of tissue compartments and histopathological features

**Figure 1:**
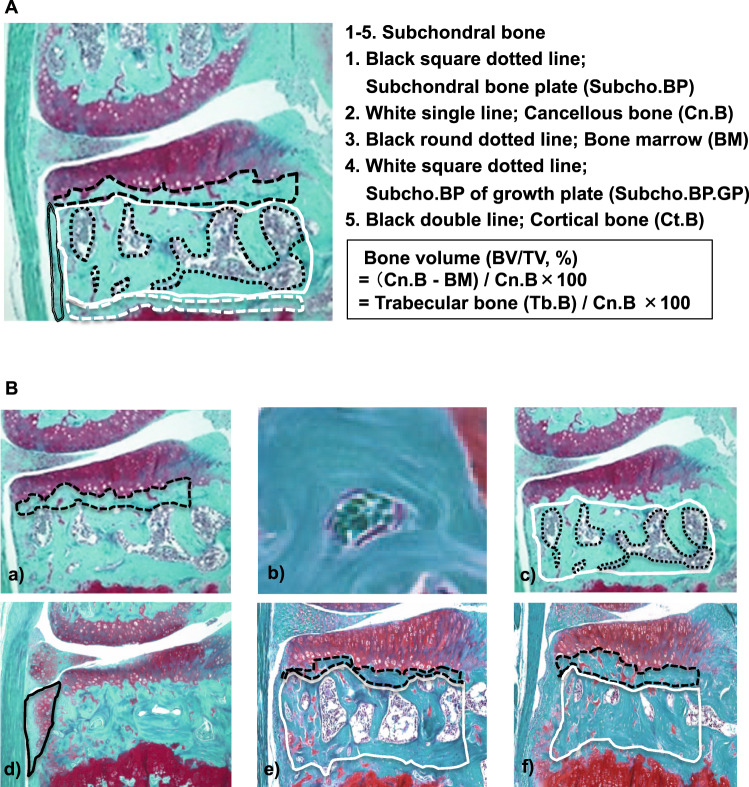
Summary of the subchondral bone scoring system. (**A**) Definition of tissue compartments and histopathological features in the subchondral bone. We distinguished specific regions in the subchondral bone, which encompasses the epiphysis. Black square dotted line; subchondral bone plate (Subcho.BP), White single line; cancellous bone (Cn.B), Black round dotted line; bone marrow, White square dotted line; Subcho.BP of growth plate (Subcho.BP.GP), Black double line; cortical bone (Ct.B). The region of Subcho.BP is defined as the region between the osteochondral junction and the BM cavity. The mean of Subcho.BP.thickness (Subcho.BP.Th) was calculated by dividing the area of Subcho.BP by the length of the bottom. Cn.B includes BM and trabecular bone (Tb.B). Since bone volume (BV/TV) reflects evaluation of Cn.B, the regions of Subcho.BP, Subcho.BP.GP and Ct.B were not included for the evaluation of BV/TV. We defined the calculation method of BV/TV as follows: Bone volume (BV/TV, %) = (Cn.B region – BM regions)*/Cn.B region ×100, * Cn.B region – BM regions = Tb.B regions. (**B**) Sample Images for each parameter of subchondral bone scoring system. Sample images for each parameter of subchondral bone scoring system and two examples which were used to calculate Subcho.BP.Th and BV/TV by using Image J with bone histomorphometry method. a) Subcho.BP: Black square dotted line shows the region of Subcho.BP. b) Angiogenesis: We identified two or more red blood cells in the luminal structure lined with endothelial cells in Subcho.BP as angiogenesis. c) BV/TV; BV/TV means the ratio of Tb.B in Cn.B. White single line shows the lesion of Cn.B. Black round dotted line shows the region of BM. d) Osteophyte; Black single line shows the osteophyte as grade 3. e) Case 1; Black square dotted line shows the region of Subcho.BP. White single line shows the region of Cn.B. We calculated as Subcho.BP.Th = 26.1 µm, BV/TV = 64%. f) Case 2; We calculated as Subcho.BP.Th = 39.1 µm, BV/TV = 86%.

For developing a subchondral bone scoring system, we defined the compartments within the osteochondral unit in histology, including articular cartilage (AC), subchondral bone plate (Subcho.BP), cancellous bone (Cn.B), cortical bone (Ct.B) and subchondral bone plate of growth plate (Subcho.BP.GP) (Fig. 1A). Cn.B included trabecular bone (Tb.B) and bone marrow (BM). Components of subchondral bone, which encompasses the epiphysis include Subcho.BP, Cn.B, Ct.B, Subcho.BP.GP.

### Definition of subchondral bone plate (Subcho.BP) and bone volume (BV/TV)

Subcho.BP represents only part of the subchondral bone. The Subcho.BP was defined in histology as the region between the osteochondral junction and the BM cavity (Fig. 1A). This area was measured by Image J, the mean of Subcho.BP.Th was calculated by dividing the area of Subcho.BP by the length of the bottom according to same methods of a previous report^15^. A reasonable measure in the histological section is to be able to know the relative three dimensional analysis from two dimensional section, using an indirect method of bone histomorphometry^48^. The indirect method is also reported less laborious and less subject to sampling bias^48^. As BV/TV reflects evaluation of Cn.B calculated by the ratio of Tb.B and BM, we did not include the Subcho.BP, Subcho.BP.GP and Ct.B for the evaluation of BV/TV. Therefore, we defined the calculation method of BV/TV as follows: Bone volume (BV/TV, %) = (Cn.B region − BM regions)*/Cn.B region × 100, (*Cn.B region − BM regions = Tb.B regions) (Fig. 1A). We calculated Subcho.BP thickness (Subcho.BP.Th) and BV/TV by using Image J with bone histomorphometry method^48,49^.

### Subchondral bone scoring system and sample images of each parameter

Parameters were selected based on a review of publications that evaluated subchondral bone changes in animal OA models^9,12,17,18,36–39,41,50–54^. In OA progression, Subcho.BP.Th usually becomes thicker^10,18,20,30^, and BV/TV increases^16,17,39^. Angiogenesis has been reported to play an important role in the onset and progression of OA^9,18,52^. Osteophytes are a main structural and early-stage OA feature^55^. Histologically, osteophytes are composed of the cartilage and bone^54^. Thus, we can detect and evaluate osteophytes at earlier phase of OA than by uCT. As mentioned above, the subchondral bone scoring system described here has three parameters, Subcho.BP consisting of the combination of Subcho.BP.Th and angiogenesis, BV/TV, and osteophytes (Fig. 1B). After quantifying each parameter except for osteophytes, they were graded on a scale of Subcho.BP 0–6; BV/TV 0–3; osteophyte 0–3 (Table 2). Minimum score is zero points, and maximal score is 12. This scoring system was established by using coronal sections stained with Safranin O. These parameters were scored in the medial tibial plateau. Angiogenesis was defined as two or more red blood cells in the luminal structure lined with endothelial cells in Subcho.BP. We consider angiogenesis is derived from bone as previous paper reported that osteoclasts infiltrate and neovascularization occurs from the bone up to cartilage^9^. For evaluation of osteophytes, we used Kamekura’s osteophyte score^53^. Two examples are shown in Fig. 1(B-e,f).

**Table 2:** Summary of Semi-quantitative Subchondral Bone Scoring System.

A. Subchondral bone plate (0–6)		thickness compared to control
0	Same as control	
1	No increase in thickness but bone-cartilage interface undulates	lesser than 110%*
2	Slight increase in thickness without angiogenesis	111–125%
3	Slight increase in thickness with a few angiogenesis	111–125%
4	Moderate increase in thickness with several angiogenesis	126–150%
5	Marked increase in thickness with several angiogenesis	151–175%
6	Major increase in thickness with several angiogenesis	greater than 176%
**B. Bone volume (BV/TV) (0–3)**		
0	No change	
1	Mild increase (BV/TV; 61–70%)	
2	Moderate increase (BV/TV; 71–85%)	
3	Marked increase (BV/TV; greater than 86%)	
**C. Osteophyte (0–3)**		
0	None	
1	Formation of cartilage-like tissue	
2	Increased in cartilaginous matrix	
3	Endochondral ossification	

Minimum score is zero, maximum score is 12.

### Histopathological assessments

All knee joints were embedded intact in paraffin after fixation in 4% Paraformaldehyde Phosphate Buffer Solution and decalcification in 10% EDTA. Knee joints were sectioned (4.5 μm) in the coronal plane through the central weight-bearing region of the anterior and posterior femorotibial joint^56^. The sections were stained with Safranin O. We analyzed three sections per mouse knee that represent the central weight bearing area of the medial and lateral femoral joints where cartilage changes occur early and become most severe. The average of the quantified parameter of the three sections was calculated. Articular cartilage was scored using the OARSI scoring system for histological assessments of OA in the mouse^24^. Osteophyte formation was evaluated according to a previously described histopathological classification system^53^. Three individuals familiar with histopathological grading of joint tissues quantified each parameter by using image J. Three sets of scores were used to assess the interclass reliability of the histological scoring system. In addition, one grader scored the same collection of sections twice (three weeks apart) to assess the intraclass reproducibility of the grading system^57^.

### Mouse models

All animal experiments were performed according to protocols approved by the institutional Animal Care and Use Committees at Hiroshima University and Scripps Research.

We evaluated the subchondral bone changes using two different OA models in mice; 1) senescence accelerated mouse (SAM)-prone 8 (SAMP8)^44–46^ as spontaneous OA model with SAM-resistant 1 (SAMR1) as the control. 2) destabilization of the medial meniscus (DMM)^47^ in C57BL/6 mice as surgical OA model. SAMP8 and SAMR1 were obtained from Japan SLC (Shizuoka, Japan), and only male mice were used in this study. Knee joints were harvested at 4, 6, 9 and 14 weeks of age to monitor spontaneous age-related OA (n = 6/group). The weight of SAMP8 mice was lower than SAMR1 mice at 14 weeks of age (Supplementary figure 1).

Experimental OA was induced in 10-week-old male C57BL/6J mice (N = 6/group) by transection of the medial meniscotibial ligament (MMTL) in the right knees as described^47^. The left (contra-lateral) knee was subjected to sham surgery for control. We also included mice that had no surgery as controls (n = 3). Mice were sacrificed 2 (n = 6), 7 (n = 6), and 56 days (n = 5) after surgery, and the knee joints were collected for analysis.

### Microcomputed tomography (µCT)

In this study, µCT was used only for validation of this histological system because our main purpose was to develop a tool that can be applied to the histological sections stained Safranin O which is most widely used in the analysis of murine OA study.

To analyze mouse tibias, images were captured under the conditions of 80 kV (tube voltage) and 100 µA (tube current) and at the resolution of 4.848 µm/voxel by a ScanXmate-D090S105 scanner (Comscantechno, Yokohama, Japan). Regions of interest were defined to measure Subcho.BP.Th and BV/TV in the medial tibial plateau corresponding to the region of interest in the histological analyses. Three-dimensional microstructural (3D-µCT) image data were reconstructed, and structural parameters were calculated using a TRI/3D-BON software (Ratoc System Engineering, Tokyo, Japan)^58^. Bone mineral density was estimated by means of phantoms with known density of hydroxyapatite, and it was shown by pseudo color images.

### Statistical analyses

Actual measurement value data are presented as mean ± standard deviation of the mean. These data were compared between SAMP8 and SAMR1 groups, and the DMM and contra-lateral groups at every time point by using Welch’s t test as a result of the normality test (Shapiro-Wilk test, p ≧0.05). Multiple comparisons among groups were performed using Dunnett’s test after-measures by one-way analysis of variance (ANOVA). Scored data are presented as median and range. These data were compared between the OA and control groups using Mann-Whitney U test for non-parametric analysis. We used Spearman’ rank correlation coefficient to analyze the correlation between the data of bone scoring system and the data of µCT measurements. The intra-rater and inter-rater reliability of Cronbach’s alpha for each parameter were tested by initials KN and KN, HK, YI. P-values less than 0.05 were considered significant. SPSS Statistics software version 21 (SPSS Inc., Chicago, IL, USA) was used for the all analyses except for Cronbach’s alpha test version 25 was used.

### Ethics approval

All animal experiments were performed according to protocols approved by the institutional Animal Care and Use Committees at Hiroshima University and Scripps Research.

## Results

### SAMP8 as spontaneous OA model

**Figure 2:**
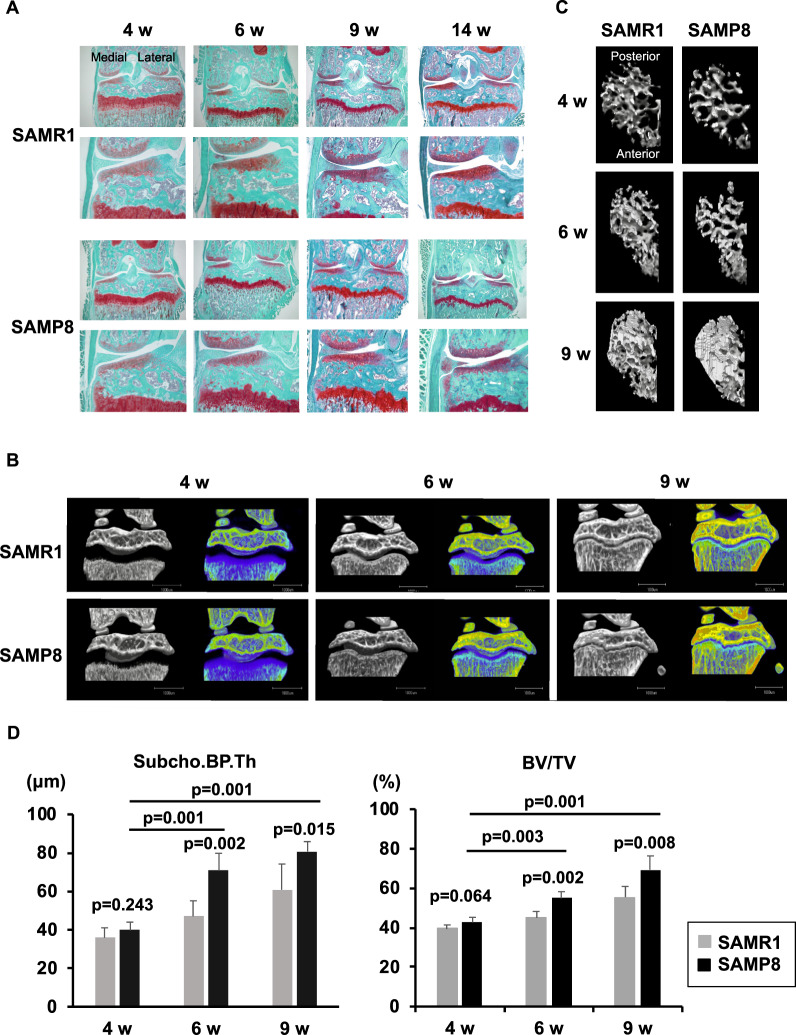
Senescence accelerated mouse (SAM)-prone 8 (SAMP8) as spontaneous OA model. (**A**) Histology (Coronal section, Safranin O stain); SAM-resistant 1 (SAMR1) is the control of SAMP8. We evaluated mice at 4, 6, 9, and 14 weeks of age. Bone changes already occurred in SAMP8 at 6 weeks of age, but not in SAMR1. In SAMP8, cartilage damage and osteophyte formation occurred by 14 weeks of age. (N = 6 each group in histology. N = 5 each group in µCT). (**B**) Images of µCT (1): We obtained µCT images from the same SAMP8 and SAMR1 mouse strains that were analyzed by the bone histology scoring system (N = 5/group). We also obtained colored 3D-µCT images using calcium phosphate. (**C**) Images of µCT (2): Figures show the 3-dimensional image looking up at the entire cancellous bone region at the medial epiphysis of the tibia. The change of the trabecular bone in medial tibial plateau is evident. (**D**) µCT data of subchondral bone thickness (Subcho.BP.Th) and bone volume (BV/TV): There were significant differences in both Subcho.BP.Th and BV/TV at 6 and 9 weeks of age between SAMP8 and SAMR1 (Welch’s t test). In addition, there were significant increase of Subcho.BP.Th and BV/TV among SAMP8 groups with aging (Dunnett’s test). * All data were presented as mean ± standard deviation of the mean. P  <  0.05 was statistically significant.

To investigate histopathological subchondral bone changes in a spontaneous OA model, the knee joints from SAMP8 and SAMR1 mice were evaluated at 4, 6, 9, and 14 weeks of age by histology (Fig. 2A). From 4 to 9 weeks of age, SAMP8 and SAMR1 had intact articular cartilage and similar proteoglycan staining. At 14 weeks of age, OA severity in SAMP8 ranged from minimal changes, such as reduction of proteoglycan to cartilage fibrillation or partial defects which were not present in SAMR1. We obtained µCT images from some of the same SAMP8 and SAMR1 mice (N = 5/group) that were analyzed by the subchondral bone histology scoring system (Fig. 2B). We also obtained µCT images in the anteroposterior direction to visualize changes in Tb.B using the knee joints before decalcification (Fig. 2C). Images represent views of the entire cancellous bone region at the medial epiphysis of the tibia. Statistically significant differences were already detected in both Subcho.BP.Th and BV/TV at 6 weeks of age between SAMP8 and SAMR1 (p = 0.002 in both Subcho.BP.Th and BV/TV). In addition, Subcho.BP.Th was getting dramatically thicker from 4 weeks to 6 and 9 weeks of age among SAM‘8 groups, and BV/TV was also increasing significantly with aging (Fig. 2D).

**Figure 3:**
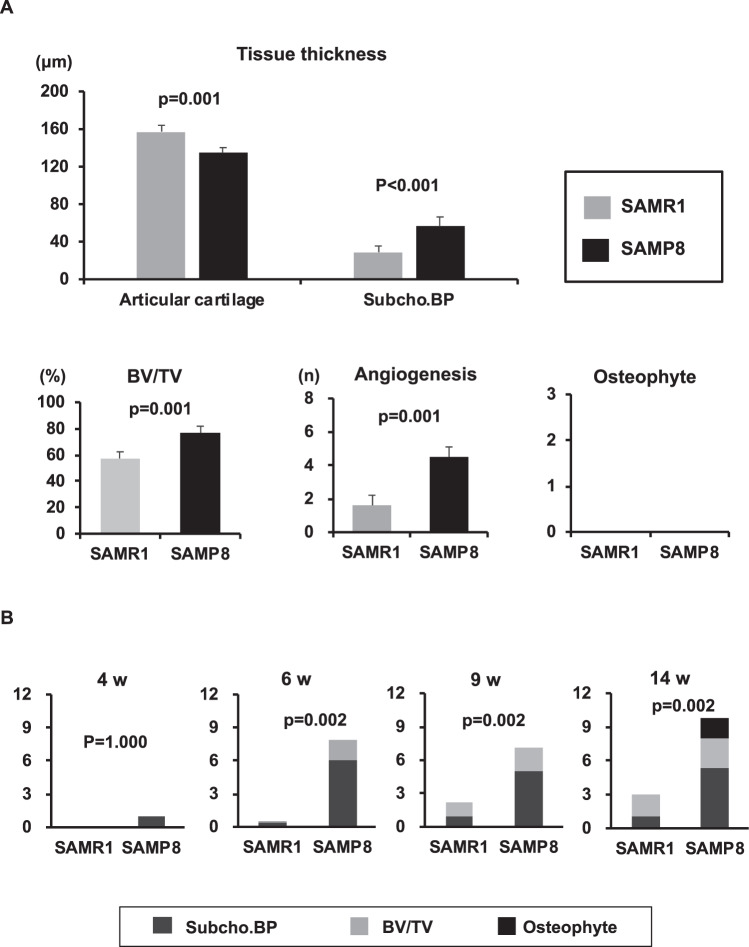
Quantification of each subchondral bone parameter at 6 weeks old and time course of subchondral bone score in spontaneous OA model. (**A**) Senescence accelerated mouse (SAM)-prone 8 and SAM-resistant 1 (SAMR1) at 6 weeks of age (n = 6). There were significant differences in the thickness of subchondral bone plate (Subcho.BP), articular cartilage (AC), and in bone volume (BV/TV) and angiogenesis in medial tibial plateau between SAMP8 and SAMR1 (Welch’s t test). There were no osteophytes in both groups. All data were presented as mean ± standard deviation of the mean. P  <  0.05 was statistically significant. (**B**) Time course of subchondral bone changes in spontaneous OA model. (**C**) The subchondral bone score was already significantly higher at 6 weeks of age in senescence accelerated mouse SAMP8 than in its control SAMR1 (Mann-Whitney U test). The subchondral bone score increased further from 6 to 14 weeks of age in SAMP8. *All data were presented as mean. P  <  0.05 was statistically significant.

In histology, several changes in subchondral bone could be detected in SAMP8 at 6 weeks of age, but not in SAMR1. At 6 weeks of age in SAMP8, Subcho.BP was thicker, angiogenesis and BV/TV were increased significantly in medial tibial plateau compared to SAMR1 (p < 0.001, 0.001, 0.001, respectively) (Fig. 3A). Subchondral bone score using this system was already dramatically higher at 6 weeks of age in SAMP8 than in SAMR1 (p = 0.002) and increased further from 6 to 14 weeks of age in SAMP8 (Fig. 3B).

### Surgical OA model

**Figure 4:**
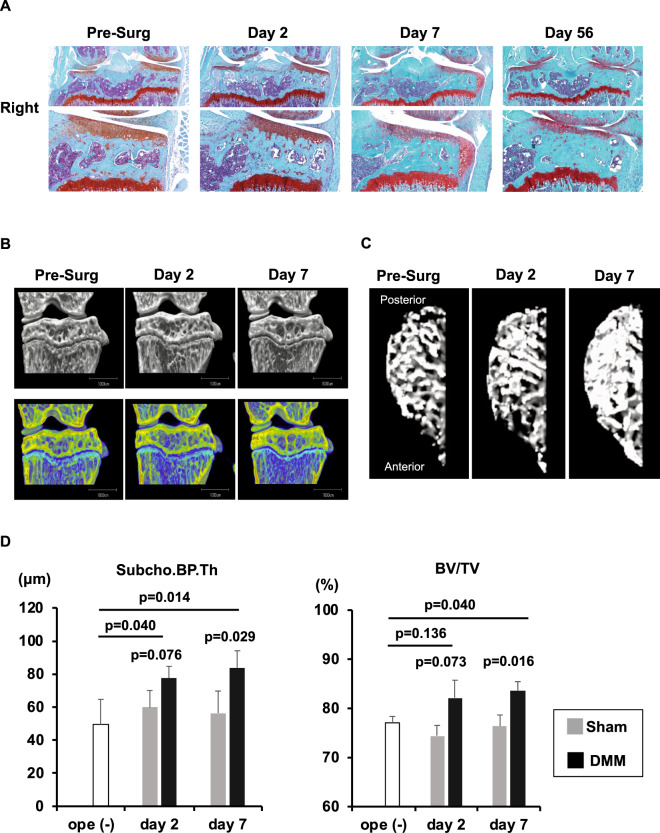
Destabilization of the medial meniscus (DMM) in C57BL/6 mice as surgical OA model. (**A**) Histology (Coronal section, Safranin O stain): We performed DMM in the right knee and sham surgery in the contra-lateral knee (Sham knee) of 10 weeks old C57BL/6 mice and evaluated post-surgery days 2, 7, and 56 (N = 6 post-surgery day 2 and 7, N = 5 post-surgery day 56). In the DMM knees, a marked difference in subchondral bone was recognized by post-surgery day 7. At the same time, chondrophytes had already appeared in all mice. There were significant differences between DMM knees (right) and Sham knees post-surgery day 7. (**B**) Images of µCT (1): We obtained µCT images of bilateral knee from the same C57BL/6 mouse strains that were analyzed by the bone histology scoring system (N = 3/group). (**C**) Images of µCT (2): The change in the trabecular bone in the medial tibial plateau is evident. (**D**) µCT data of subchondral bone plate thickness (Subcho.BP.Th) and bone volume (BV/TV); There were significant differences in both Subcho.BP.Th and BV/TV on post-surgery day 7 between DMM knees and Sham knees (Welch’s t test). In DMM knees, the Subcho.BP.Th was getting thicker until post-surgery day 2 and BV/TV was increasing in until post-surgery day 7 (Dunnett’s test) * All data were presented as mean ± standard deviation of the mean. P  <  0.05 was statistically significant.

To investigate histopathological subchondral bone changes in a surgically induced OA model, we performed DMM in the right knee (DMM knee) and sham surgery in the contra-lateral knee (Sham knee) of 10 weeks old C57BL/6 mice and evaluated post-surgery days 2, 7 and 56 (N = 6 post-surgery day 2 and 7, N = 5 post-surgery day 56) (Fig. 4A). We obtained µCT images of bilateral knees from some of the same C57BL/6 mice that were analyzed by the bone histology scoring system (N = 3/group) (Figs. 4B, 4C). There were significant differences in both Subcho.BP.Th and BV/TV post-surgery day 7 between DMM knees and Sham knees (p = 0.029 in Subcho.BP.Th, p = 0.016 in BV/TV, respectively). In DMM knees, the Subcho.BP.Th was getting dramatically thicker until post-surgery day 2 and BV/TV was increasing significantly until post-surgery day 7 (Fig. 4D).

**Figure 5:**
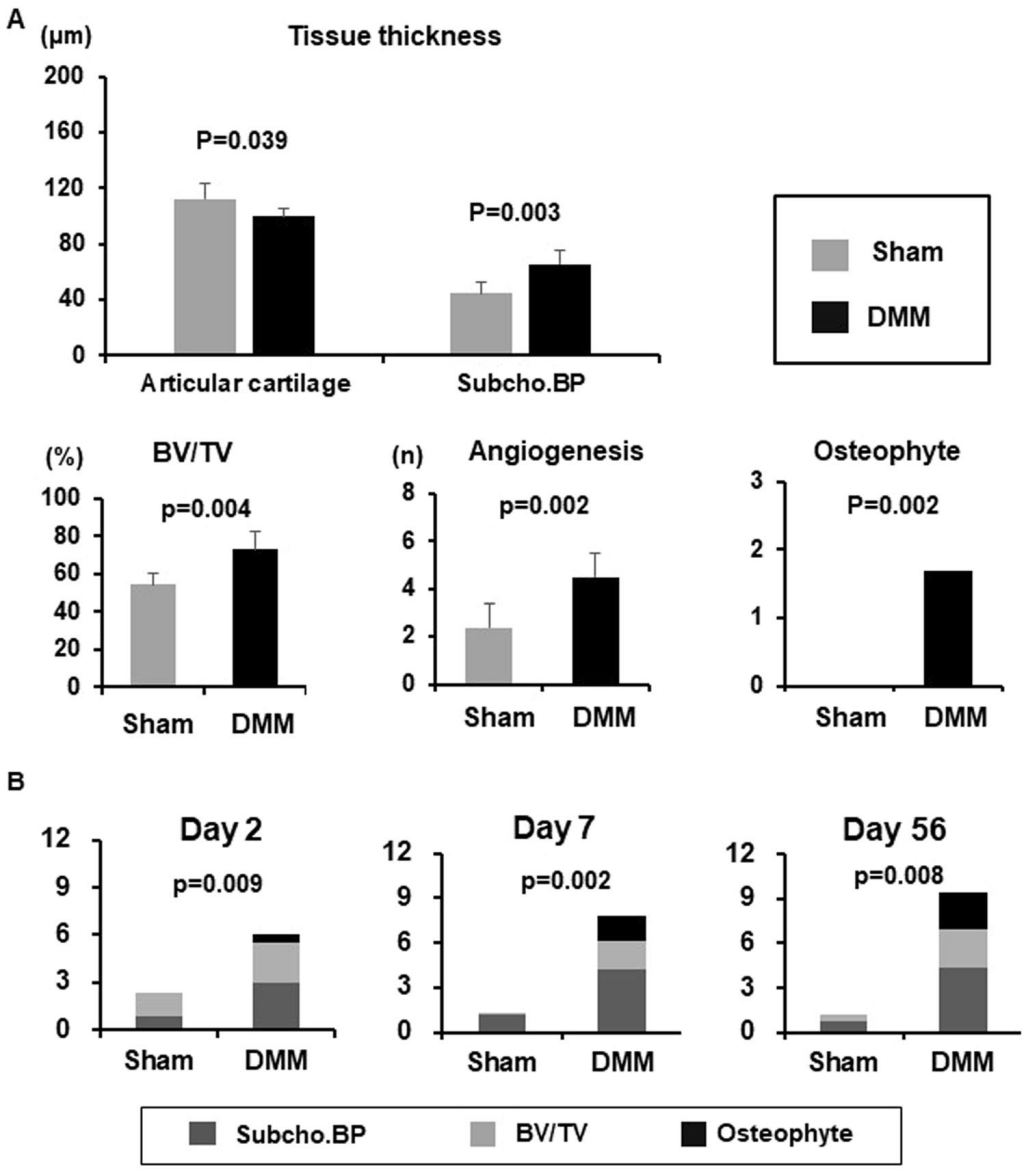
Quantification of each subchondral bone parameter on post-surgery day 7 and time course of subchondral bone scores in surgical OA model. (**A**) Destabilization of the medial meniscus (DMM) and contra-lateral (Sham) knees post-surgery day 7 (n = 6). While there was no significant difference between DMM and Sham knees in the thickness of the articular cartilage (AC) of medial tibial plateau yet, but there were already significant differences in the subchondral bone plate (Subcho.BP). Bone volume (BV/TV) and angiogenesis were also increased significantly in DMM knees compared to Sham knees (Welch’s t test). In addition, osteophytes had already appeared in all DMM knees on day 7, but none in Sham knees (Mann-Whitney U test). *All data except for osteophyte score were presented as mean ± standard deviation of the mean, osteophyte score was presented as only mean. P  <  0.05 was statistically significant. (**B**) Time course of subchondral bone changes in the surgical OA model. Subchondral bone score was already higher in the DMM knees compared to the Sham knees on post-surgery day 2 (p = 0.009). Furthermore, the differences increased further on post-surgery day 7 (p = 0.002). * All data were presented as mean. P  <  0.05 was statistically significant.

In histology, several changes in subchondral bone could be also detected in DMM knees on post-surgery day 7, but not in Sham knees. In DMM knees, Subcho.BP were thicker, angiogenesis and BV/TV were increased significantly in medial tibial plateau post-surgery day 7 compared to Sham knees (p = 0.003, 0.002, 0.004, respectively). In addition, osteophytes (grade 1 or 2) had already appeared in all mice by day 7, but none in Sham knees (Fig. 5A). Notably, subchondral bone score using this system was already dramatically higher in DMM knees than Sham knees post-surgery day 2 (p = 0.009) and the differences increased further on post-surgery day 7 (p = 0.002) (Fig. 5B).

### Comparison of bone and cartilage scoring systems

Tables 3 and 4 shows the comparison of subchondral bone score using this system and OARSI cartilage score. In the spontaneous OA model in SAMP8 mice, there was already a significant difference in the subchondral bone scores compared to SAMR1 at 6 weeks of age (p = 0.002). However, at 6 weeks of age, there were no differences in OARSI cartilage total scores which became higher at 14 weeks of age. In the surgical OA model, there were already significant differences in subchondral bone scores post-DMM day 2 (p = 0.009). However, cartilage damage in tibia was not evident until post-surgery day 7. The OARSI total score was only modestly elevated post-DMM day 7 with greater increase on post-DMM day 56.

**Table 3:** Comparison of bone grades and OARSI^*1^ cartilage score in spontaneous OA model.

Spontaneous OA study			4 w	6 w	9 w	14 w
OARSI cartilage score (0–12)	SAMR1^*2^	median (range)	0 (0–1)	1.0 (1)	1.0 (0.5–1)	0 (0)
	SAMP8^*3^		0 (0–1)	1.0 (1)	1.0 (0.5–2)	4.5 (0–7)
		p value	1.000	1.000	0.731	0.065
Subchondral bone score (0–12)	SAMR1	median (range)	0 (0)	0 (0–2)	2.0 (1–3)	3.0 (2–4)
	SAMP8		0.5 (0–3)	8.0 (7–8)	7.0 (4–9)	10.0 (8–12)
		p value	1.000	0.002	0.002	0.002

Scored date were presented as median and range. We compared the data between the right knee of SAMP8 and SAMR1 using Mann-Whitney U test. N = 6, all time points both of SAMP8 and SAMR1. *1: Osteoarthritis research society international, *2: senescence accelerated mouse (SAM)-prone 8, *3: SAM-resistant 1.

**Table 4:** Comparison of bone grades and OARSI^*1^ cartilage score in the surgical OA model.

Surgical OA study			Pre-ope	Day 2	Day 7	Day 56
OARSI cartilage score (0–12)	Sham^#1^	median (range)	0 (0)	0 (0)	1.0 (0–1)	0.5 (0–1)
	DMM^*2^			0 (0)	1.5 (1–3)	3.0 (2.5–5)
		p value	—	1.000	0.009	0.008
Subchondral bone score (0–12)	Sham	median (range)	0.5 (0–1)	2.0 (1–4)	1.0 (0–3)	1.0 (0–3)
	DMM			6.5 (4–8)	7.5 (5–11)	9 (8–11)
		p value	—	0.009	0.002	0.008

Scored data were presented as median and range. We compared the data between DMM knees (right) and Sham knees (contra-lateral) using Mann-Whitney U test. N = 6 at pre-DMM, post-DMM day 2, and day7. N = 5 at post-DMM Day56. *1: Osteoarthritis research society international, *2: destabilization of the medial meniscus, #1: sham surgery in the contra-lateral knee.

### Validation of bone scoring system

**Figure 6:**
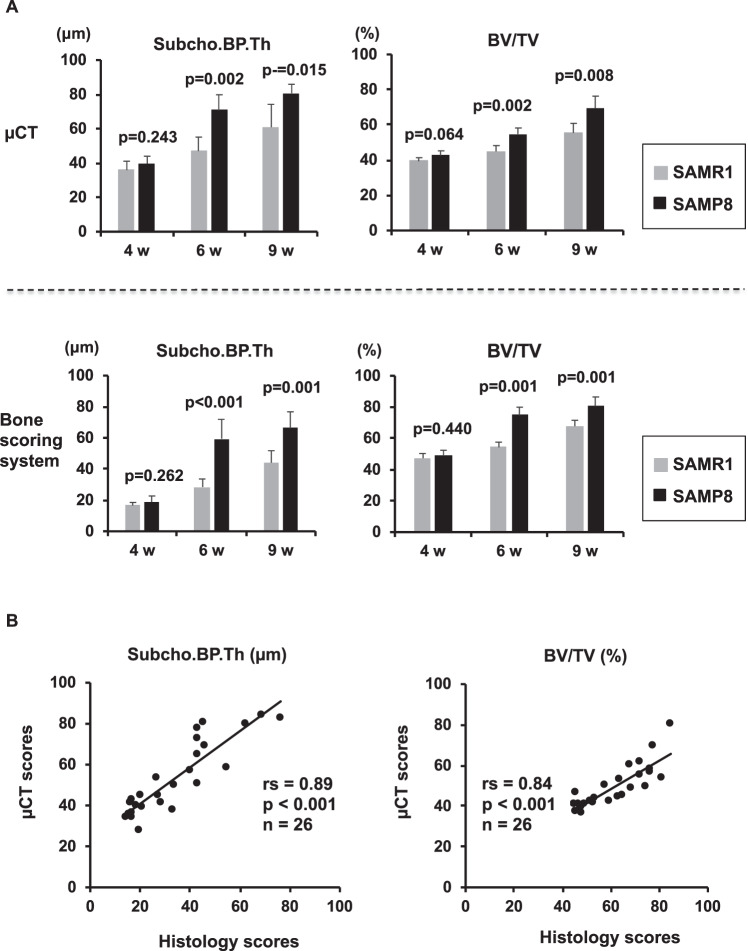
Validation of bone scoring system by µCT. (**A**) Comparison of histological bone scoring system and µCT measurements in senescence accelerated mouse (SAM) model. Quantified data of subchondral bone thickness (Subcho.BP.Th) and bone volume (BV/TV) in bone scoring system were quite similar to those of µCT. Subcho.BP.Th and BV/TV of SAM-prone 8 (SAMP8) at 6 and 9 weeks were significantly higher than in SAM-resistant 1 (SAMR1) in both bone scoring system and µCT (Welch’s t test). (**B**) Correlation between histological bone scoring system and µCT measurements. There was a strong correlation in both Subcho.BP.Th and BV/TV between the histological bone scoring system and µCT (rs = 0.89 in Subcho.BP.Th, rs = 0.84 in BV/TV, P < 0.001, n = 26, Spearman’ rank correlation coefficient).

In this histological scoring system, the intra-rater reliabilities of Cronbach alpha for each parameter were more than 0.906, the inter-rater reliabilities were 0.943 (Supplementary table 1, 2). Furthermore, we obtained µCT images from the same SAMP8 and SAMR1 mouse strains that were analyzed by the bone histological scoring system. Data from the histological scoring system for Subcho.BP.Th and BV/TV were quite similar to those of µCT (Fig. 6A). Subcho.BP.Th and BV/TV at 6 and 9 weeks of age in SAMP8 were significantly higher than those of SAMR1 in both bone scoring system and µCT. There was a strong correlation both of Subcho.BP.Th and BV/TV between this histological scoring system and µCT (R = 0.89, 0.84, respectively, P < 0.001) (Fig. 6B). In the surgical OA model, the results were similar to the spontaneous OA model with good correlations between histological scoring and µCT (R = 0.85, 0.81). Thus, this system could be a useful tool for evaluating the subchondral bone changes in murine OA models.

## Discussion

Here we developed a histopathological subchondral bone scoring system, which can evaluate the major early changes in subchondral bone in murine models of knee OA. We selected three important and widely used parameters; 1) Subcho.BP consisting of the combination of Subcho.BP.Th and angiogenesis, 2) BV/TV, 3) osteophytes. Among them, Subcho.BP.Th, angiogenesis, and BV/TV are semi-quantified and scored. Subcho.BP changes start first as undulations at the bone and cartilage interface as previously reported^14^, then, Subcho.BP.Th increases, and angiogenesis becomes apparent. BV/TV also increases gradually during early phase of OA. Subsequently, osteophyte score increased. When we used our semi-quantitative subchondral bone scoring system, subchondral bone scores in both OA models were significantly increased already at early phase. In addition, subchondral bone scores were increased much earlier than OARSI cartilage scores in both OA models. In this regard, we were able to evaluate details of how subchondral bone changes developed at the very early phase of OA by using this histopathological scoring system in two murine OA models. Further, our histological evaluation system was validated and not to inferior to µCT.

Injury-induced mouse models of OA^53,59^ are widely used. However, there are few spontaneous mouse models of OA^60^. SAM strains have been successfully developed by selective inbreeding of the AKR/J strain of mice^45,46^. They are divided into P type (prone) showing accelerated aging/short life span and R type (resistant) showing normal aging. Among of SAMP, SAMP8 is a mouse model developing aging-related diseases such as neurodegenerative disorder accompanied by deteriorations of memory and learning ability with aging^44^. Knee joints from 14-week-old SAMP8 mice showed reduced Safranin O staining which indicates proteoglycan loss, and roughening and fibrillation of articular surface, especially in medial tibial plateaus. At 23 weeks of age, SAMP8 mice showed severe OA with partial-thickness defects on the medial tibial plateau, and these changes were significantly more severe with aging (Miyaki S *et al*.: unpublished observations). Thus, these results indicate that subchondral bone changes in SAMP8 occur before the onset of cartilage degeneration.

Injury-induced mouse models of OA such as DMM is widely used as secondary OA model. In this model, lesions are primarily located on the central weight bearing areas of tibial plateau and femoral condyles and lesion severity increases as a function of time^20,37,47^. Our recent report^16^ and the present study showed that the subchondral bone mass significantly increased at day 7 after DMM by µCT analysis. However, our new scoring system detected significant histological differences with increased subchondral bone scores between DMM knees and control knees at post-surgery day 2 by only evaluating specimens stained were stained with Safranin O. Thus, this quantitative scoring system is also very sensitive for evaluating the early subchondral bone changes in the DMM model and can be applicable readily.

It has been reported that angiogenesis is an important process in OA progression^9,18,52,61–63^. Vascular ingrowth into the cartilage occurs via the pores in subchondral bone, subsequently, osteoblasts infiltrate and start to deposit bone, resulting in sclerosis^9^. Zhen *et al*. reported TGF-β1 is activated in the subchondral bone in response to abnormal mechanical loading and stimulate increases in the number of MSCs, and osteoprogenitors in the bone marrow, which lead to aberrant bone formation and angiogenesis, promoting for OA progression^18^. This study used CT-based microangiography of the tibia subchondral bone and quantification of subchondral bone vessel volume and vessel number^18^. However, due to it is high cost, it is not practical to use microangiography for all studies focusing on subchondral bone in OA. In this regard, our scoring system is feasible because only Safranin O stained sections are needed. Furthermore, we were able to evaluate subchondral bone and BV/TV by using already established bone morphometric methods with Image J soft and this method was not inferior to µCT. This scoring system is not only readily applicable but also useful to accurately evaluate subchondral bone changes.

Recently, the concept of crosstalk of cartilage-bone has received much attention^10^. Subchondral bone changes before the onset of cartilage degeneration in the present study suggest that subchondral bone play an important role in OA pathogenesis. Therefore, the development of agents for OA treatments and prevention require an understanding of the condition of subchondral bone, before and after intervention. In addition, it may be even more important to determine the timing of the intervention depending on the condition of the joint tissues including subchondral bone. This scoring system for histopathological assessment provided a standardized and easily applicable technique to analyze bone changes in murine models of OA without the need for special equipment such as µCT. The information on bone changes can be collected from the same sections that are prepared to assess cartilage changes. We expect that this scoring system will be used further validated by other laboratories and will improve the quality and scope of studies including the effect due to therapeutic agents using murine models of OA. The study has the following limitations. We evaluated subchondral bone only using decalcified sections. Non-decalcified sections may be better for evaluation of subchondral bone. However, since decalcified sections stained with Safranin O are commonly used and specifically useful for detecting cartilage changes, we chose three parameters that could be evaluated. Finnila *et al*. compared human decalcified histological sections with Subcho.BP.Th on µCT. Since µCT includes calcified cartilage, the value was higher than that of histology, but the correlation was good^30^. We analyzed three sections per mouse knee that represent the central weight bearing area of the medial and lateral femoral joints using indirect methods of bone histomorphometry. Our results were similar to the previous results^30^ and correlation was also good.

In some mouse OA models, there is subchondral bone loss during early phases. In our scoring system this can be captured by the calculation of Subcho.BP.Th and/or BV/TV. In both OA models in our study, Subcho.BP.Th in the MTP was increased compared to respective control groups. We infer this difference occurred because the region of Subcho.BP was strictly defined, and the measurement was accurate.

## Conclusions

A new semi-quantitative histological scoring system is readily applicable for evaluating the early changes in aging and OA-affected murine subchondral bone. This system may be a useful tool for further studies of subchondral bone in murine OA models.

## Supplementary information

Supplementary information.

## Data Availability

The datasets used and/or analyzed during the current study are available from the corresponding author on reasonable request.
