# Supplementary material for: Histological scoring system for subchondral bone changes in murine models of joint aging and osteoarthritis
Source: Sci Rep. 2020 Jun 22;10:10077. doi: 10.1038/s41598-020-66979-7 (PMC7308327; doi:10.1038/s41598-020-66979-7)
Supplement: Supplementary file 1 — Supplementary information. [file 41598_2020_66979_MOESM1_ESM.docx]

**Histological scoring system for subchondral bone changes in murine models of joint aging and osteoarthritis**

Keita Nagira^1, 2^, Yasunari Ikuta^1, 3^, Masahiro Shinohara^4^, Yohei Sanada^3, 5^, Takenori Omoto^3^, Haruhisa Kanaya^2^, Tomoyuki Nakasa^3^, Masakazu Ishikawa^3^, Nobuo Adachi^3^, Shigeru Miyaki^3, 5#^ and Martin Lotz^1#^

^1^Department of Molecular Medicine, Scripps Research, La Jolla, California

^2^ Department of Orthopaedic Surgery, Tottori University, Tottori, Japan

^3^Department of Orthopaedic Surgery, Graduate School of Biomedical & Health Sciences, Hiroshima University, Hiroshima, Japan

^4^Department of Rehabilitation for the Movement Functions, National Rehabilitation Center for Persons with Disabilities, Saitama, Japan

^5^Medical Center for Translational and Clinical Research, Hiroshima University Hospital, Hiroshima, Japan

^#^ Co-corresponding author

Correspondence to:

Shigeru Miyaki, Ph.D.

Medical Center for Translational and Clinical Research, Hiroshima University Hospital

E-mail: [miyaki@hiroshima-u.ac.jp](about:blank)

Martin Lotz, M.D.

Department of Molecular Medicine, Scripps Research

E-mail: [mlotz@scripps.edu](about:blank)

**
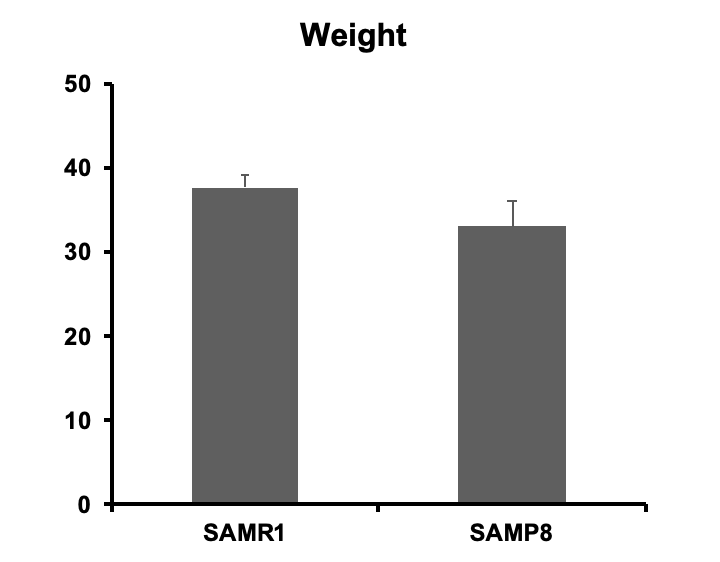
**

**Supplementary figure 1.** The comparison of weight at 14 weeks of age in spontaneous OA models.

The weight of Senescence accelerated mouse (SAM)-prone 8 (SAMP8) was significantly lower than SAM-resistant 1 (SAMR1) at 14 weeks old (p=0.003).

* These data were presented as mean ± standard deviation of the mean. Statistical analysis was assessed by Welch’s t test.

**Supple table 1.** Actual measurement value, score, and intra-rater reliability of Cronbach alpha for each parameter at 6 weeks in spontaneous OA model.

|  |  |  |  | Nagira 　1st | Nagira 2nd | Intra-rater  reliability | 95% CI^*1^ |
| --- | --- | --- | --- | --- | --- | --- | --- |
| Subchondral bone plate thickness  (µm) | SAMP8^*2^ | value | mean (±SD) | 46.8 　(10.2) | 54.9 　(8.6) | 0.921 | 0.725-0.977 |
|  | SAMR1^*3^ |  |  | 28.3 　(6.5) | 23.5 　(3.3) |  |  |
| Angiogenesis　 (n) | SAMP8 | value | mean (±SD) | 3.9 　　(0.7) | 3.6 　　(0.3) | 0.953 | 0.835-0.986 |
|  | SAMR1 |  |  | 1.8 　　(0.5) | 1.1 　　(0.7) |  |  |
| Bone Volume  (%) | SAMP8 | value | mean (±SD) | 74.8 　(5.3) | 64.6 　(5.9) | 0.906 | 0.673-0.973 |
|  | SAMR1 |  |  | 54.3　 (5.9) | 53.7 　(5.3) |  |  |
| Osteophyte | SAMP8 | score | median (range) | 0 (0) | 0 (0) | - | - |
|  | SAMR1 |  |  | 0 (0) | 0 (0) |  |  |

An alpha factor of 0.8 or higher is considered consistent. *1 CI: confidence intervals. *2 Subcho.BP.Th: subchondral bone plate thickness, *3 SAMP8: senescence accelerated mouse-prone 8, *4 SAMR1: senescence accelerated mouse-resistant 1.

**Supple table 2.** Actual measurement value, score, and inter-rater reliability of Cronbach alpha for each parameter post-surgery day 7 in surgical OA model.

|  |  |  |  | Kanaya | Nagira | Ikuta | Inter-rater  reliability | 95% CI^*1^ |
| --- | --- | --- | --- | --- | --- | --- | --- | --- |
| Subcho.BP.Th^*2^ (µm) | DMM^*3^ | value | mean (±SD) | 65.4 (11.6) | 65.6 (10.2) | 61.5 (9.5) | 0.943 | 0.848-0.982 |
|  | Sham^#1^ |  |  | 48.6 (10.4) | 44.4 (8.8) | 43.1 (10.5) |  |  |
| Angiogenesis　 (n) | DMM | value | mean (±SD) | 3.3 　(0.7) | 4.8 　(1.2) | 4.4　 (0.6) | 0.951 | 0.871-0.985 |
|  | Sham |  |  | 1.7 　(0.8) | 2.4　 (0.6) | 1.8 　(0.5) |  |  |
| Bone Volume  (%) | DMM | value | mean (±SD) | 71.4 (9.1) | 72.8 (10.8) | 72.0 (11.8) | 0.960 | 0.893-0.987 |
|  | Sham |  |  | 59.0 (7.5) | 54.3 (5.9) | 60.6 (7.7) |  |  |
| Osteophyte | DMM | score | median (range) | 2.0　　(1-2) | 2.0 　　(1-2) | 2.0　　(1-2) | 0.985 | 0.942-0.993 |
|  | Sham |  |  | 0 　　(0) | 0 　　 (0) | 0 　　 (0) |  |  |

An alpha factor of 0.8 or higher is considered consistent. *1 CI: confidence intervals, *2 Subcho.BP.Th: subchondral bone plate thickness, *3 DMM: destabilization of the medial meniscus, #1 Sham: sham surgery in the contra-lateral knee.
